# Spectroscopic Identification and Characterization of Three Rotamers of *m*-Ethoxyphenol: Combined REMPI, MATI, and Quantum Chemical Study

**DOI:** 10.3390/ijms27104166

**Published:** 2026-05-07

**Authors:** Xiateng Qin, Yan Zhao, Keke Zhang, Rui Wang, Zhonghua Ji, Changyong Li, Suotang Jia

**Affiliations:** 1 State Key Laboratory of Quantum Optics Technologies and Devices, Institute of Laser Spectroscopy, Shanxi University, Taiyuan 030006, China; 18734558738@163.com (X.Q.); zhangkeke323@163.com (K.Z.); 19861241857@163.com (R.W.); jzh@sxu.edu.cn (Z.J.); tjia@sxu.edu.cn (S.J.); 2 Department of Physics and Electronics Engineering, Jinzhong University, Jinzhong 030619, China; zhaoy@jzxy.edu.cn; 3 Collaborative Innovation Center of Extreme Optics, Shanxi University, Taiyuan 030006, China

**Keywords:** *m*-ethoxyphenol, rotamer, REMPI spectroscopy, MATI spectroscopy, hole-burning, adiabatic ionization energy, vibrational assignment

## Abstract

Rotational isomers (rotamers) of substituted aromatic molecules exhibit distinct physicochemical properties that are fundamental to understanding their reactivity and biological functions. However, resolving individual rotamers spectroscopically remains challenging due to their similar transition energies and overlapping spectral features. Herein, we report the conformer-specific identification and characterization of three stable rotamers of *m*-ethoxyphenol using a combination of resonance-enhanced multiphoton ionization (REMPI), hole-burning (HB) spectroscopy, and mass-analyzed threshold ionization (MATI) techniques, complemented by high-level quantum chemical calculations at the B3PW91/aug-cc-pVTZ and G4 levels of theory. The S_1_ ← S_0_ electronic origins of rotamers I, IV, and III were determined to be 35,966 ± 2, 36,031 ± 2, and 36,198 ± 2 cm^−1^, respectively, while their corresponding adiabatic ionization energies (IEs) were precisely measured as 64,574 ± 5, 64,122 ± 5, and 64,994 ± 5 cm^−1^. The vibrational spectra of both the S_1_ excited state and the D_0_ cationic ground state were assigned, with most active modes corresponding to in-plane benzene ring vibrations. Structural analysis reveals that the benzene ring undergoes slight expansion upon S_1_ ← S_0_ excitation and contraction upon D_0_ ← S_1_ ionization, while the overall molecular geometry remains remarkably similar across all three electronic states, a key factor underlying the excellent agreement between experimental and simulated Franck–Condon spectra. Comparison with *m*-methoxyphenol demonstrates that the stronger electron-donating ability of the ethoxy group leads to systematically lower excitation and ionization energies. The distinct spectroscopic fingerprints established herein provide a definitive reference for identifying specific *m*-ethoxyphenol rotamers in future studies of this molecule and its complexes.

## 1. Introduction

Phenolic derivatives constitute a fundamental class of compounds with widespread importance in biological systems, pharmaceutical chemistry, and materials science. As the base chromophore of the essential amino acid tyrosine, phenol and its substituted derivatives have attracted sustained research interest due to their pivotal role in ultraviolet (UV) absorption processes that underlie photochemical and photobiological phenomena [1,2]. Among these derivatives, alkoxyphenols—bearing both hydroxyl and alkoxy substituents—are particularly intriguing because both functional groups can participate in chemical reactions through electron donation to the aromatic ring [3]. The rotational flexibility about the single bonds connecting these substituents to the benzene ring gives rise to multiple rotational isomers, or rotamers, which can coexist in chemical samples and exhibit distinct physicochemical properties [4,5].

The existence of multiple rotamers poses both opportunities and challenges. While rotamers may display markedly different chemical reactivity and biological activity [6], their similar molecular structures lead to electronic excitation energies (E_1_) and ionization energies (IEs) that differ by only tens to hundreds of wavenumbers [7]. Conventional spectroscopic techniques such as infrared (IR), Raman, or fluorescence emission spectroscopy generally lack the resolution to disentangle the overlapping spectral features arising from different rotamers in a mixture. This limitation necessitates the application of conformer-specific spectroscopic methods combined with supersonic molecular beam cooling, which isolates individual rotamers in a low-temperature environment and suppresses conformational interconversion [8,9].

Resonance-enhanced multiphoton ionization (REMPI) spectroscopy coupled with time-of-flight (TOF) mass spectrometry provides a powerful approach for recording vibronic spectra of mass-selected species [10,11,12,13,14,15]. However, even with mass selectivity, spectra of mixtures containing multiple rotamers can be congested due to overlapping vibronic transitions, as different rotamers share the same mass. Hole-burning (HB) spectroscopy addresses this limitation by optically selecting individual conformers, enabling unambiguous assignment of vibronic bands to specific rotamers [16,17,18,19,20,21]. For investigating cationic state properties, mass-analyzed threshold ionization (MATI) spectroscopy offers unique advantages [22,23,24,25,26]. This technique combines the high resolution of zero-kinetic energy (ZEKE) photoelectron spectroscopy with mass selectivity [27], allowing precise determination of adiabatic IEs and vibrational frequencies of selected rotamer cations. The MATI method has proven particularly valuable for studying isotopes [28,29], molecular clusters [30,31,32], and radicals [33] due to its mass-resolving capability.

*meta*-Ethoxyphenol (*m*-ethoxyphenol, C_6_H_4_(OH)(OC_2_H_5_)) presents an interesting case for conformational analysis. With both hydroxyl and ethoxy groups attached to the benzene ring in a *meta* relationship, multiple stable rotamers can arise from different orientations of these substituents. The ethoxy group itself possesses two rotationally flexible bonds (O–CH_2_ and CH_2_–CH_3_), further enriching the conformational landscape. Previous studies on related molecules have established important precedents. Ullrich et al. thoroughly characterized three rotamers of *m*-methoxyphenol using ZEKE and hole-burning spectroscopy [34,35], while Wilke et al. employed rotationally resolved electronic spectroscopy to elucidate the conformational space of the same molecule [36]. For *para*-substituted analogues, Zheng et al. investigated *p*-ethoxyphenol rotamers using MATI spectroscopy [37], and Li et al. studied *p*-methoxyphenol cations [38]. These investigations demonstrate that alkoxy substitution systematically lowers excitation and ionization energies, with the magnitude of the shift correlating with the electron-donating strength of the substituent.

In this work, we present a comprehensive spectroscopic investigation of *m*-ethoxyphenol rotamers using complementary techniques: potential energy surface (PES) scanning to identify stable conformers, REMPI spectroscopy combined with HB to resolve vibronic spectra of individual rotamers, and MATI spectroscopy to precisely determine adiabatic IEs and characterize cationic vibrations. High-level quantum chemical calculations at the B3PW91/aug-cc-pVTZ and G4 levels support experimental assignments and provide insights into structural changes accompanying electronic excitation and ionization. By comparing our results with those for *m*-methoxyphenol [34,35,36], we elucidate the influence of ethoxy versus methoxy substitution on molecular properties. The conformer-specific spectroscopic fingerprints established herein serve as essential references for future studies of *m*-ethoxyphenol-containing systems, including molecular clusters and complexes relevant to atmospheric chemistry and materials science.

## 2. Results

### 2.1. Theoretical Conformational Landscape of m-Ethoxyphenol

**Figure 1:**
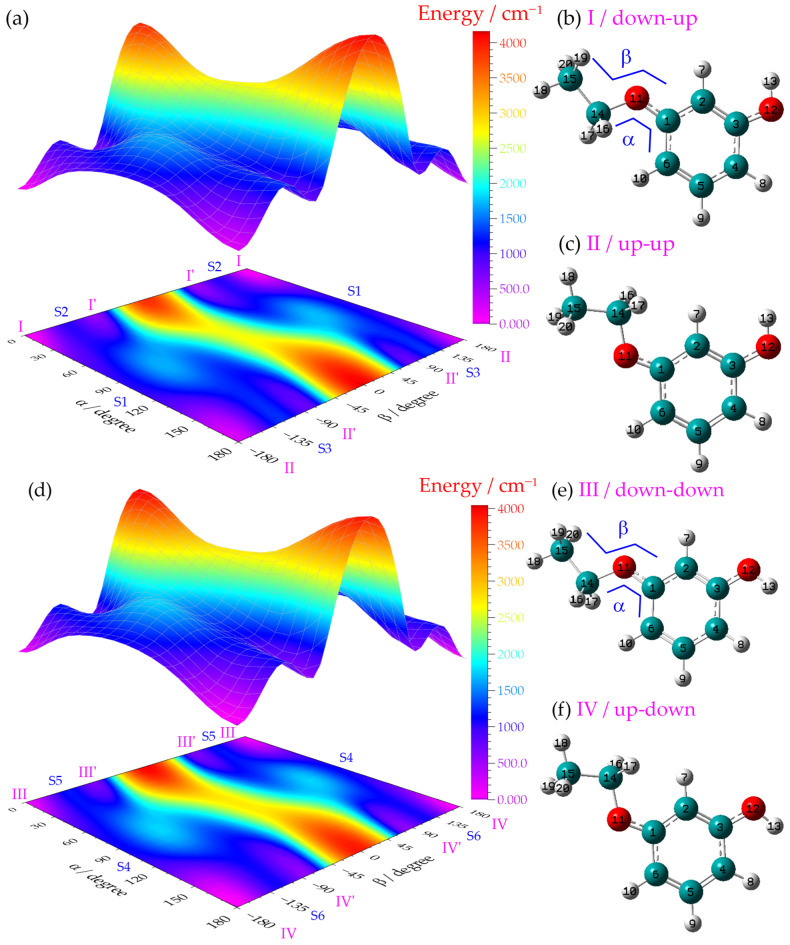
Potential energy surfaces (**a**,**d**) obtained by scanning two torsional angles, α, β, of the ethoxy group while fixing the hydroxyl orientation (up or down). On each surface, eight local minima are identified. The two lowest-energy rotamers on surface (**a**) are labeled as I (**b**) and II (**c**), and their counterparts with the methyl group protruding out of the ring plane are denoted as I’ and II’, respectively. Similarly, the two lowest-energy conformers on surface (**d**) are labeled as III (**e**) and IV (**f**), with their out-of-plane methyl variants designated as III’ and IV’. ∠β. The relative energies of the six saddle points S1–S6 are 1113, 1124, 1359, 1082, 1182, and 1085 cm^−1^, respectively, relative to rotamer IV. The four conformers I, II, III, and IV, optimized at the B3LYP/6-311++G(d,p) level (see Table 1 in the main text), have relative energies of 58, 226, 90, and 0 cm^−1^, respectively. Atomic labels are provided in the four stable conformers shown.

The ground-state potential energy surface (PES) of *m*-ethoxyphenol was systematically explored by scanning two key torsional angles of the ethoxy group: α (∠C6–C1–O11–C14) and β (∠C1–O11–C14–C15), as defined in Figure 1. It is well known that the hydroxyl group exhibits only two stable in-plane orientations (0° and 180°). A 180° rotation around the C–O bond from one orientation to the other entails an energy barrier of over 1000 cm^−1^ (see Figure S1), rendering this process negligible at room temperature. For the up (0°) and down (180°) orientations of the hydroxyl group, PES calculations were performed at the B3LYP/6-311++G(d,p) level. Figure 1a,d show the PES obtained by scanning the two torsional angles of the ethoxy group with the OH group in the up and down orientations, respectively. Eight local minima were identified on each PES. The two lowest-energy minima on PES (a) correspond to conformers I (b) and II (c). Their analogous structures with the methyl group oriented out of the aromatic plane are denoted as I’ and II’, respectively. Similarly, the two dominant low-lying conformers on PES (d) are labeled III (e) and IV (f), along with their out-of-plane methyl analogs III’ and IV’. Atomic labeling is shown for the four representative stable rotamers illustrated herein.

To facilitate comparison with the well-characterized *m*-methoxyphenol system, the naming convention for rotamers I–IV has been kept consistent with that in the referenced literature [34,35,36,39,40]. Table 1 presents the relative zero-point energies (ZPEs) of all rotamers calculated using four different theoretical methods: B3LYP/6-311++G(d,p), B3PW91/6-311++G(d,p), B3LYP/aug-cc-pVTZ, and B3PW91/aug-cc-pVTZ. Rotamer IV consistently exhibits the lowest ZPE across all methods, in excellent agreement with the conformational preferences observed for *m*-methoxyphenol [34,36].

Rotamers I’, II’, III’, and IV’ lie significantly higher in energy (≥569 cm^−1^ above rotamer IV). According to the Maxwell–Boltzmann distribution, the ground-state populations of these high-energy rotamers are negligible under supersonic jet expansion conditions [41]. Consequently, subsequent discussion focuses on the four low-energy rotamers I–IV. Based on the behavior of *m*-methoxyphenol, where rotamer II was not experimentally observable due to its higher energy and steric hindrance [34,36], we anticipated that only rotamers I, III, and IV would be detectable in our experiments.

### 2.2. Vibronic and Hole-Burning Spectra

**Figure 2:**
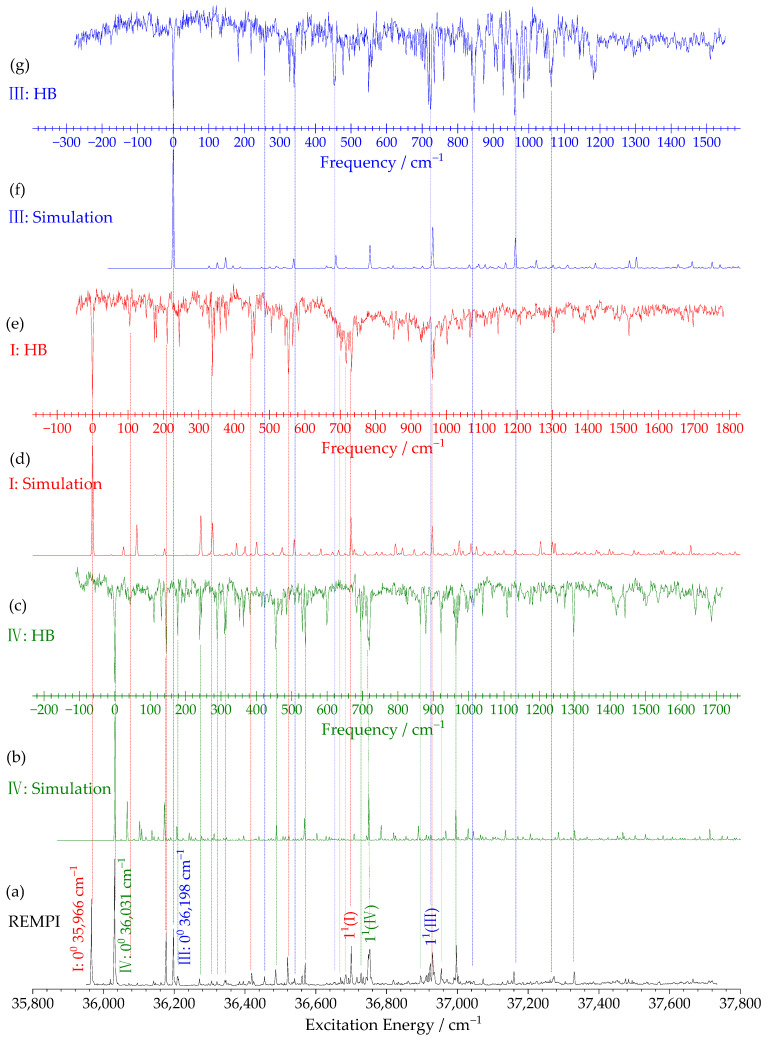
(**a**) Two-color R2PI spectrum near the S_1_ ← S_0_ 0^0^ electronic transition. (**b**,**d**,**f**) Simulated S_1_ ← S_0_ vibronic spectra of rotamers IV, I, and III, respectively, at the TD-B3PW91/aug-cc-pVTZ level (scaling factor 0.98). (**c**,**e**,**g**) Hole-burning spectra of rotamers IV, I, and III, respectively. The bands at 35,966 ± 2, 36,031 ± 2, and 36,198 ± 2 cm^−1^ are assigned as the S_1_ ← S_0_ origins of rotamers I, IV, and III. The x-axis represents excitation energy (cm^−1^) for (**a**) and vibrational frequency shift (cm^−1^) relative to the origin band for (**b**–**g**).

Figure 2 presents the two-color resonance-enhanced two-photon ionization (2C-R2PI) spectrum of *m*-ethoxyphenol in the region near its S_1_ ← S_0_ electronic transition. The ionization laser was fixed at 33,393 cm^−1^ to avoid fragmentation of larger clusters into the monomer mass channel. The 2C-R2PI technique offers the advantage of accurately reflecting S_1_ ← S_0_ transition intensities by increasing the ionization laser intensity to ionize most molecules in the resonantly excited S_1_ state.

The HB spectra (Figure 2c,e,g) confirm the presence of three distinct rotamers. With the probe laser fixed at the origin of a selected rotamer, the burn laser wavelength is scanned. When the burn laser is resonant with any energy level of the probed rotamer, that rotamer is depleted, leading to a significant decrease in the probe signal. The resulting dips correspond to the vibrational energy levels of the targeted rotamer. All prominent vibronic features in the REMPI spectrum exhibit corresponding holes, unequivocally demonstrating that three stable rotamers of *m*-ethoxyphenol coexist in the supersonic molecular beam.

The assignment of these three rotamers to specific structures (I, IV, and III) was achieved through comprehensive consideration of multiple theoretical and experimental criteria:

1. Ground-state energies: The relative ZPEs (Table 1) predict rotamer IV as the most stable, with rotamers I and III lying slightly higher (48–90 cm^−1^). Using the Boltzmann distribution, the expected intensity ratio of the 0_0_^0^ bands is IV:I:III ≈ 1:0.8:0.77, consistent with the experimental REMPI intensities.
2. Franck–Condon simulations: Figure 2b,d,f display the simulated S_1_ ← S_0_ vibronic spectra of rotamers IV, I, and III, calculated at the TD-B3PW91/aug-cc-pVTZ level. The excellent agreement between experimental and simulated spectra for each rotamer provides strong support for the assignment.
3. Ionization energies: As detailed in Section 2.3, the experimentally determined IEs and their relative ordering (IV < I < III) match theoretical predictions.
4. Rotamer II absence: Simulated Franck–Condon factors for rotamer II were found to be extremely small, indicating negligible S_1_ ← S_0_ transition probability (See Figure S2). This parallels observations for resorcinol or its cluster with CO and water [42,43] and *m*-methoxyphenol [34,36], where the analogous high-energy rotamer (with both substituents oriented unfavorably) is not observed experimentally due to steric hindrance.

Table 2 lists the observed S_1_ state vibrational bands, their frequency shifts relative to the origin, calculated frequencies, and tentative vibrational assignments for each rotamer. Vibrational modes are labeled according to Wilson’s notation for benzene derivatives as adapted by Varsanyi and Szoke [44,45]. Most active vibrations correspond to in-plane benzene ring deformation modes and substituent-sensitive bending motions.

Notably, the benzene ring breathing vibration (mode 1^1^) appears at distinctly different frequencies for the three rotamers: 732, 719, and 724 cm^−1^ for rotamers I, IV, and III, respectively. This sensitivity to the relative orientation of the substituents underscores the utility of HB spectroscopy for distinguishing closely related rotamers.

### 2.3. Cationic Spectra and Ionization Energies

Prior to the MATI experiments, adiabatic IEs of the four low-energy rotamers were calculated using the high-level composite methods G4 and CBS-QB3, which are known to provide reliable energetics for small to medium-sized molecules. Table 3 presents the relative energies of the S_0_ and D_0_ states, the calculated IEs, and the experimental IEs, together with the theoretical errors.

The calculations consistently predict rotamer IV as the most stable in the S_0_ state, while rotamer II becomes the most stable in the D_0_ state—a reversal analogous to that observed for resorcinol [42,43,46] and *m*-methoxyphenol [34,36]. The calculated IEs follow the order II < IV < I < III, with rotamer II showing the lowest IE.

**Figure 3:**
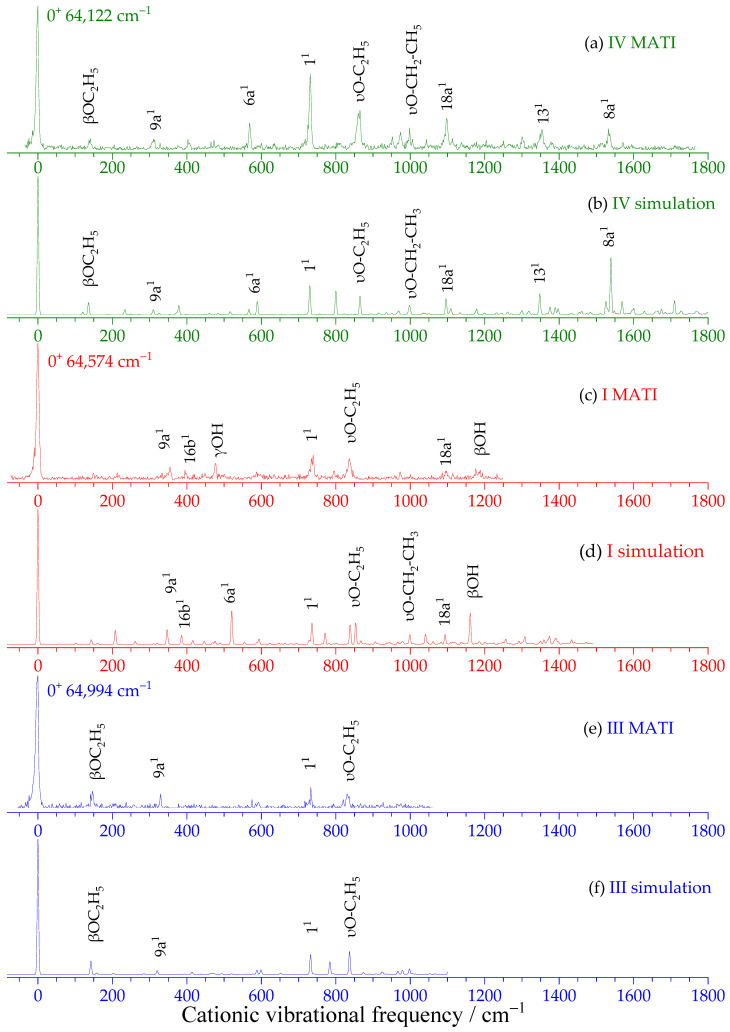
MATI spectra of *m*-ethoxyphenol rotamers IV (**a**), I (**c**) and III (**e**) via the S_1_ 0^0^ level, along with theoretical simulations (**b**,**d**,**f**) at the B3PW91/aug-cc-pVTZ level with harmonic frequencies scaled by 0.98. The adiabatic ionization energies (0^+^ bands) are determined as 64,122 ± 5, 64,574 ± 5 and 64,994 ± 5 cm^−1^ for rotamers IV, I, and III, respectively.

Figure 3 displays the MATI spectra of rotamers IV (a), I (c) and III (e), recorded via their respective S_1_ ← S_0_ 0^0^ origins (36,031, 35,966 and 36,198 cm^−1^). The simulated D_0_ ← S_1_ vibronic spectra at the B3PW91/aug-cc-pVTZ level (scaling factor 0.98) are shown in Figure 3b, Figure 3d and Figure 3f for rotamers IV, I and III, respectively.

From the sharp 0^0^ bands in the MATI spectra, the adiabatic IEs were precisely determined as: Rotamer IV: 64,122 ± 5 cm^−1^; Rotamer I: 64,574 ± 5 cm^−1^; and Rotamer III: 64,994 ± 5 cm^−1^. The measured IE trend (IV < I < III) is fully consistent with G4 calculations. All deviations remain below 0.3%, demonstrating reliable computational performance for ionization energy evaluation. The distinct IE values serve as definitive fingerprints for identifying specific *m*-ethoxyphenol rotamer cations.

Table 4 presents the measured vibration assignments for the D_0_-state cations. The spectra exhibit rich vibrational structure, reflecting the influence of conformational differences on cationic vibrational properties.

## 3. Discussion

### 3.1. Structural Changes upon Electronic Excitation and Ionization

The excellent agreement between experimental and simulated vibronic spectra (Figure 2 and Figure 3) indicates that the theoretical methods employed (B3PW91/aug-cc-pVTZ for S_0_ and D_0_ states; TD-B3PW91/aug-cc-pVTZ for S_1_ state) reliably describe the molecular and spectral properties of *m*-ethoxyphenol. Tables S1–S3 present the optimized geometric parameters for rotamers IV, I and III in the S_0_, S_1_, and D_0_ states, respectively, along with changes upon electronic excitation and ionization.

Several important trends emerge from the structural analysis:

1. Upon S_1_ ← S_0_ excitation: The benzene ring undergoes slight expansion, with C–C bond lengths increasing by 0.013–0.039 Å. This is consistent with the π* ← π electronic excitation, which weakens the bonding character of the ring carbon–carbon bonds. Concurrently, the C–O bonds (C1–O11 and C3–O12) shorten slightly (by 0.006–0.011 Å), indicating increased double-bond character due to electron redistribution.
2. Upon D_0_ ← S_1_ ionization: The benzene ring contracts, with C–C bond lengths decreasing by 0.019–0.043 Å relative to the S_1_ state. The removal of an electron restores and even strengthens the π-bonding character of the cationic ring. The C–O bonds shorten substantially (by 0.030–0.044 Å), reflecting the increased electron density withdrawal toward the positively charged ring. The O11–C14 bond elongates (by 0.033–0.038 Å), suggesting weakening of this bond in the cation.
3. Substituent geometry: The C6–C1–O11 bond angle deviates by approximately 4° from the ideal sp^2^ angle of 120°, suggesting a weak non-covalent interaction between the lone-pair electrons of the ethoxy oxygen and the adjacent hydrogen atoms on the benzene ring. This interaction induces a slight geometric distortion at the substitution site—a feature common to all three rotamers.
4. Hydroxyl group orientation: The angular geometry of the hydroxyl group remains nearly unchanged upon S_1_ ← S_0_ excitation (Δ < 0.5°), but undergoes a small reorientation (≈3.5°) upon D_0_ ← S_1_ ionization, reflecting altered electron density distribution in the cationic state.

Remarkably, the overall molecular geometry remains very similar across all three electronic states for each rotamer. This structural similarity underlies the excellent Franck–Condon overlap observed in both S_1_ ← S_0_ and D_0_ ← S_1_ transitions, explaining the dominance of the origin bands and the good agreement between experimental and simulated spectra.

Although the optimized torsional angles of the ethoxy group do not show significant variations across the S_0_, S_1_, and D_0_ electronic states (as summarized in Tables S1–S3), the torsional potential energy surface (see Figure 1) exhibits multiple local minima separated by low energy barriers, indicating that the ethoxy group is highly flexible. Consequently, the low-frequency torsional motion of the ethoxy group cannot be adequately described by the harmonic approximation, and a global scaling factor derived from high-frequency vibrational modes is expected to have limited accuracy for these torsional modes. This anharmonicity is a direct consequence of the shallow and multi-minimum nature of the potential energy surface governing the ethoxy rotation. The present torsional scan provides explicit evidence of this anharmonic behavior, and we therefore caution that the vibrational assignments involving the ethoxy group in Table 2 and Table 4 should be considered as tentative, pending more rigorous theoretical treatments (e.g., full anharmonic calculations) in future studies.

### 3.2. Comparison with m-Methoxyphenol: Substituent Effects

Table 5 compares the E_1_ and IE values of *m*-ethoxyphenol (this work) with those of the corresponding rotamers of *m*-methoxyphenol [34,36].

Both E_1_ and IE of *m*-ethoxyphenol are consistently lower than those of the corresponding *m*-methoxyphenol rotamers. The E_1_ values differ by only 3–8 cm^−1^, while the IE differences are substantial (619–654 cm^−1^). This trend directly reflects the slightly stronger electron-donating ability of the ethoxy group (OC_2_H_5_) relative to methoxy (OCH_3_): the longer alkyl chain enhances the inductive effect, increasing electron density on the benzene ring and the entire molecular framework. Consequently, less energy is required to promote a π electron to the π* orbital (S_1_ ← S_0_ excitation) and to remove a valence electron (ionization) in *m*-ethoxyphenol.

The much larger effect on IE compared to E_1_ can be rationalized by considering the nature of the two processes. S_1_ ← S_0_ excitation involves promotion of an electron within the π system, while ionization completely removes an electron. The cation experiences the full inductive effect of the ethoxy group, whereas the neutral excited state benefits only partially from the enhanced electron donation.

### 3.3. Absence of Rotamer II

The calculations predict rotamer II to lie 198–226 cm^−1^ above rotamer IV in the S_0_ state (Table 1), which would suggest a non-negligible population under supersonic jet conditions. Yet, no spectroscopic features attributable to rotamer II were observed in either REMPI or HB experiments. This absence parallels observations for *m*-methoxyphenol [34,36], resorcinol [42,43,46], m-dimethoxybenzene [47] and m-diethoxybenzene [40].

Two factors likely contribute to the non-observability of rotamer II:

1. Franck–Condon factors: The simulated Franck–Condon factors for the S_1_ ← S_0_ transition of rotamer II were found to be extremely small (see Supporting Information Figure S2). This is in sharp contrast to rotamers I, III, and IV, where the origin band dominates the spectrum. Such a low Franck–Condon factor for the origin transition would make rotamer II undetectable under typical experimental conditions.
2. Steric hindrance: In rotamer II, both the hydroxyl hydrogen and the ethoxy group are oriented “upward” (pointing toward the same side of the ring), creating steric repulsion between the ethoxy and hydroxyl groups. This steric strain elevates the ground-state energy and may also distort the excited-state geometry, further reducing Franck–Condon factors.

The absence of rotamer II underscores an important caveat in conformational analysis: the relative intensities of origin bands in electronic spectra do not necessarily reflect ground-state populations, as differing Franck–Condon factors can dramatically affect transition probabilities.

## 4. Materials and Methods

### 4.1. Experimental Methods

*meta*-Ethoxyphenol (≥98% purity, Shanghai TCL Chemical Co., Ltd., Shanghai, China) was used without further purification. The sample was heated to approximately 150 °C to achieve sufficient vapor pressure and seeded in 3 bar of krypton carrier gas. The gas mixture was expanded into a vacuum through a pulsed nozzle (General Valve, Series 9, 0.5 mm orifice, Parker Hannifin Corporation, Cleveland, OH, USA) operating at 10 Hz. The resulting supersonic molecular beam passed through a skimmer (1 mm diameter) located 15 mm downstream from the nozzle, entering the ionization chamber where it intersected perpendicularly with the laser beams. Typical background pressures were ~10^−4^ Pa in the source chamber and ~10^−6^ Pa in the ionization chamber.

Two tunable dye lasers (Sirah, CBR-D-24 and Precision Scan-D, Sirah GmbH, Göttingen, Germany) pumped by a Q-switched Nd:YAG laser (Quantel, Qsmart 850, Les Ulis, Essonne, France) provided the UV radiation for two-color resonant two-photon excitation. The appropriate laser dyes (Rhodamine 6G, Coumarin 153, Exciton Inc., Dayton, OH, USA) were selected based on the expected S_1_ ← S_0_ excitation and ionization energy ranges. The output of both dye lasers was frequency-doubled using BBO crystals. A delay/pulse generator (Stanford Research Systems, DG535, Sunnyvale, CA, USA) controlled the timing between the two laser pulses, typically set to 0–10 ns for an optimal two-color signal.

For REMPI experiments, the ion extraction plates were configured as follows: the first plate was grounded, while the second and third plates (separated by 0.7 cm) were biased with pulsed electric fields of +500 V and +400 V, respectively, to accelerate photoionized ions toward the microchannel plate (MCP) detector.

For MATI experiments, a two-pulse field ionization scheme was employed [16,17]. Approximately 18 ns after the laser pulses, a pulsed electric field of −0.55 V cm^−1^ was applied to reject prompt ions produced by direct photoionization. After a delay of about 11.8 μs, a second pulsed electric field of +143 V cm^−1^ was applied to field-ionize molecules in high Rydberg states. The resulting threshold ions were then accelerated and passed through a 48 cm field-free drift tube before detection by the MCP detector. An electrostatic lens assembly was used to focus ions from different spatial positions, enhancing signal intensity.

Ion signals were processed by a multichannel scaler (Stanford Research Systems, SR430, Sunnyvale, CA, USA), accumulating data over 300 laser shots per data point. Laser wavelengths were calibrated using a wavemeter (HighFinesse WS-7, HighFinesse GmbH, Offenburg, Germany).

For hole-burning experiments, the burn laser was fired ~200 ns before the probe laser [40,48]. The probe wavelength was fixed at a selected REMPI peak (e.g., the electronic origin), while the pump wavelength was scanned. When the burn laser wavelength is resonant with any energy level of the probed rotamer, it leads to a significant decrease in the probe signal. Ions generated by the burn laser were rejected by appropriate timing and gating of the detector. The resulting dips correspond to the vibrational energy levels of the targeted rotamer. More experimental details are available in our previous publications [40,41,48,49,50].

### 4.2. Theoretical Methods

All quantum chemical calculations were performed using the GAUSSIAN 16 program package [51]. Potential energy surface scanning of the ethoxy group torsional angles α (∠C6–C1–O11–C14) and β (∠C1–O11–C14–C15) was conducted at the B3LYP/6-311++G(d,p) level, scanning both angles in 10° steps. This level provides an excellent balance of computational efficiency and accuracy for conformational exploration. For identified stable rotamers, geometry optimizations and harmonic vibrational frequency calculations were performed at higher levels. The neutral ground state S_0_ and the cationic ground state D_0_ were calculated using the B3PW91 hybrid functional [52,53] with the aug-cc-pVTZ basis set [54]. The first electronically excited state S_1_ was calculated using time-dependent DFT (TD-DFT) at the same B3PW91/aug-cc-pVTZ level [55]. All calculated harmonic vibrational frequencies were scaled by a factor of 0.98 to correct for systematic errors arising from basis set incompleteness, neglect of electron correlation, and vibrational anharmonicity [56].

Adiabatic ionization energies were also calculated using the composite methods G4 [57] and CBS-QB3 [58], which are known to provide reliable thermochemical data for small to medium-sized molecules. The IE was obtained as the difference in zero-point corrected total energies between the optimized cation D_0_ and the corresponding neutral S_0_. Vibronic spectra for S_1_ ← S_0_ and D_0_ ← S_1_ transitions were simulated within the Franck–Condon approximation, including Duschinsky rotation and Herzberg–Teller effects where appropriate [59,60]. Simulated spectra were convoluted with Gaussian line shapes (FWHM = 3.6 cm^−1^ for S_1_ spectra, 4.8 cm^−1^ for D_0_ spectra) to facilitate comparison with experimental data.

## 5. Conclusions

In this work, we have systematically characterized the rotamers of *m*-ethoxyphenol using a combination of high-resolution spectroscopic techniques and quantum chemical calculations. The main findings are summarized as follows:

Conformational identification: Three stable rotamers of *m*-ethoxyphenol were unambiguously identified in a supersonic molecular beam using REMPI and hole-burning spectroscopy. Based on ground-state energies, Franck–Condon simulations, and ionization energy measurements, these were assigned as rotamers I, IV, and III following the nomenclature established for *m*-methoxyphenol. The fourth low-energy rotamer (II) was not experimentally observed, attributed to unfavorable Franck–Condon factors for its S_1_ ← S_0_ transition and a lower ground-state population.

The electronic excitation energies for the S_1_ ← S_0_ origin transitions were precisely determined as 35,966 ± 2 cm^−1^ for rotamer I, 36,031 ± 2 cm^−1^ for rotamer IV, and 36,198 ± 2 cm^−1^ for rotamer III. Accurate adiabatic ionization energies (IEs) were obtained via MATI spectroscopy, yielding values of 64,574 ± 5 cm^−1^ for rotamer I, 64,122 ± 5 cm^−1^ for rotamer IV, and 64,994 ± 5 cm^−1^ for rotamer III. The distinct IE values serve as definitive fingerprints for identifying specific *m*-ethoxyphenol rotamer cations.

Complete vibrational assignments were established for both the S_1_ excited state and the D_0_ cationic ground state for all three rotamers. Most active modes correspond to in-plane ring vibrations and substituent-sensitive bending motions. The benzene ring breathing mode (1^1^) exhibits characteristic frequencies that distinguish both rotamers and electronic states.

Quantum chemical calculations reveal that the benzene ring undergoes slight expansion upon S_1_ ← S_0_ excitation and contraction upon D_0_ ← S_1_ ionization, while the overall molecular geometry remains remarkably similar across all three electronic states. This structural similarity underlies the excellent Franck–Condon overlap observed experimentally.

The conformer-specific spectroscopic data presented herein provide a comprehensive foundation for understanding the photophysical and photochemical properties of *m*-ethoxyphenol. These results will facilitate future studies of this molecule and its derivatives, including hydrogen-bonded clusters and complexes relevant to atmospheric chemistry and materials science.

## Figures and Tables

**Table 1:** Calculated relative zero-point energies (ZPEs) of the *m*-ethoxyphenol rotamers in the S_0_ ground state (cm^−1^) ^a^.

Rotamer	B3LYP/6-311++G(d,p)	B3PW91/6-311++G(d,p)	B3LYP/aug-cc-pVTZ	B3PW91/aug-cc-pVTZ
I/down-up	58	61	47	48
II/up-up	226	226	200	198
III/down-down	90	90	66	65
IV/up-down	0	0	0	0
I’	644	620	639	628
II’	839	813	823	810
III’	678	652	663	649
IV’	593	569	596	585

^a^ The ground-state ZPE values of rotamer IV calculated by the four methods are as follows: B3PW91/6-311++G(d,p): −461.096815 Hartree; B3LYP/6-311++G(d,p): −461.280991 Hartree; B3PW91/aug-cc-pVTZ: −461.142351 Hartree; B3LYP/aug-cc-pVTZ: −461.327201 Hartree.

**Table 2:** Experimental and theoretical vibrational frequencies (cm^−1^) of the *m*-ethoxyphenol rotamers in the S_1_ excited state, with tentative vibration assignments ^a^.

I			IV			III			Assignment ^b^
Energy	Shift	Calc.	Energy	Shift	Calc.	Energy	Shift	Calc.	
35,966	0	0	36,031	0	0	36,198	0	0	0^0^
			36,176	145	140				βOC_2_H_5_
36,177	211	204							10a^1^
			36,210	179					βOC_2_H_5_δOC_2_H_5_
			36,271	239	238				10b^1^
						36,455	257		τCH_3_
			36,320	289					βOC_2_H_5_^2^
			36,346	315					βOC_2_H_5_^2^δOC_2_H_5_
36,305	339	339				36,538	340	339	9a^1^
36,417	451	463	36,486	455	457	36,652	454	457	6b^1^
36,519	553	570	36,570	539	536	36,747	549	553	6a^1^
36,698	732	731	36,750	719	718	36,922	724	730	1^1^
			36,953	922	935				18b^1^
36,927	961	960	36,995	964	964	37,158	960	962	12^1^
			37,330	1299	1300				13^1^

^a^ Experimental values are shifts from the origin bands at 35,966 ± 2, 36,031 ± 2, and 36,198 ± 2 cm^−1^ for the respective rotamers. Theoretical vibrational frequencies at the TD-B3PW91/aug-cc-pVTZ level, scaled by 0.98. ^b^ Vibrations of the substituents: ν, stretching vibration; β, in-plane bending; γ, out-of-plane bending; τ, CH_3_ rotation; δ, other bending.

**Table 3:** Calculated relative energies of the S_0_ and D_0_ states and ionization energies of the *m*-ethoxyphenol rotamers from G4 and CBS-QB3 calculations (Unit: cm^−1^) ^a,b^.

Isomer	S_0_ Relative Energy		D_0_ Relative Energy		IE				
	G4	CBS-QB3	G4	CBS-QB3	Exp.	G4	Error	CBS-QB3	Error
I	50	50	718	714	64,574	64,373	−201	65,116	542
II	207	203	0	0		63,497		64,249	
III	70	65	1183	1112	64,994	64,817	−177	65,500	506
IV	0	0	198	187	64,122	63,902	−220	64,639	517

^a^ S_0_ state energies of rotamer IV: G4(0 K) = −461.052005 Hartree, CBS-QB3(0 K) = −460.530971 Hartree. ^b^ D_0_ state energies of rotamer II: G4(0 K) = −460.761746 Hartree, CBS-QB3(0 K) = −460.237304 Hartree.

**Table 4:** Experimental and theoretical vibrational frequencies (cm^−1^) of the *m*-ethoxyphenol rotamer cations, together with their tentative assignments ^a,b^.

I		IV		III		Assignment ^b^
Exp.	Calc.	Exp.	Calc.	Exp.	Calc.	
		141	136	146	142	βOC_2_H_5_
355	347	311	310	330	320	9a^1^
395	396					16b^1^
476	476					γOH
		568	566			6a^1^
739	736	731	730	733	732	1^1^
836	823	865	865	830	837	νO-C_2_H_5_
		998	998			νO-CH_2_-CH_3_
1098	1094	1098	1096			18a^1^
1176	1161					βOH
		1354	1347			13^1^
		1532	1539			8a^1^

^a^ Calculated values are obtained from the B3PW91/aug-cc-PVTZ calculations scaled by 0.98. ^b^ Vibrations of the substituents: ν, stretching vibration; β, in-plane bending; γ, out-of-plane bending.

**Table 5:** Comparison of S_1_ ← S_0_ excitation energies (E_1_) and adiabatic ionization energies (IE) for *m*-ethoxyphenol and *m*-methoxyphenol rotamers (cm^−1^).

Rotamer	*m*-Ethoxyphenol ^a^		*m*-Methoxyphenol ^b^		ΔE_1_	ΔIE
	E_1_	IE	E_1_	IE		
I	35,966	64,574	35,974	65,228	−8	−654
IV	36,031	64,122	36,034	64,741	−3	−619
III	36,198	64,994	36,202	65,648	−4	−654

^a^ This work. ^b^ Refs. [34,36].

## Data Availability

The original contributions presented in this study are included in the article/Supplementary Material. Further inquiries can be directed to the corresponding authors.

## Supplementary Materials

The following supporting information can be downloaded at: https://www.mdpi.com/article/10.3390/ijms27104166/s1.

## References

[1] Martinez S.J., Alfano J.C., Levy D.H. (1992). Rotationally resolved fluorescence excitation spectra of phenol and 4-ethylphenol in a supersonic jet. J. Mol. Spectrosc..

[2] Song K., Hayes J.M. (1989). Supersonic jet spectra of p-alkylphenols. J. Mol. Spectrosc..

[3] Patwari G.N., Doraiswamy S., Wategaonkar S. (2000). Spectroscopy and IVR in the S 1 State of Jet-Cooled p -Alkoxyphenols. J. Phys. Chem. A.

[4] Fidy J., Laberge M., Ullrich B., Polgar L., Szeltner Z., Gallay J., Vincent M. (2001). Tryptophan rotamers that report the conformational dynamics of proteins. Pure Appl. Chem..

[5] Dian B.C., Longarte A., Winter P.R., Zwier T.S. (2004). The dynamics of conformational isomerization in flexible biomolecules. I. Hole-filling spectroscopy of N-acetyl tryptophan methyl amide and N-acetyl tryptophan amide. J. Chem. Phys..

[6] Meyer E.A., Castellano R.K., Diederich F. (2003). Interactions with aromatic rings in chemical and biological recognition. Angew. Chem. Int. Ed. Engl..

[7] Breen P.J., Bernstein E.R., Secor H.V., Seeman J.I. (1989). Spectroscopic observation and geometry assignment of the minimum energy conformations of methoxy-substituted benzenes. J. Am. Chem. Soc..

[8] Muller-Dethlefs K., Schlag E.W. (1991). High-Resolution Zero Kinetic Energy (ZEKE) Photoelectron Spectroscopy of Molecular Systems. Annu. Rev. Phys. Chem..

[9] Müller-Dethlefs K., Schlag E.W. (1998). Chemical Applications of Zero Kinetic Energy (ZEKE) Photoelectron Spectroscopy. Angew. Chem. Int. Ed..

[10] Ketkov S.Y., Tzeng S.Y., Rychagova E.A., Kalakutskaya L.V., Fuss M., Braunschweig H., Tzeng W.-B. (2019). Rydberg state mediated multiphoton ionization of (η7-C7H7)(η5-C5H5)Cr: DFT-supported experimental insights into the molecular and electronic structures of excited sandwich complexes. Phys. Chem. Chem. Phys..

[11] Owusu-Ansah E., Cairns E., Shi Y. (2017). Characterization of Si atomic transitions using pulsed electric discharge and resonance-enhanced multiphoton ionization techniques. J. Anal. At. Spectrom..

[12] Silva W.R., Fitian M., Yang D.-S. (2022). Probing La and Ce Excited-State Reactivity with Resonant Two-Photon Ionization Spectroscopy. J. Phys. Chem. A.

[13] Zhang Z., Wang Z., Wang Q., Ma X., Wang Z., Hua Z., Yao G., Yang X., Sun Z., Qin Z. (2024). Photoionization cross sections measurements of the excited states of lutetium and ytterbium in the near threshold region. J. Chem. Phys..

[14] Hua Z., Deng J., Sun Z., Yang X., Qin Z., Zheng X. (2025). Spectroscopic signatures of the S0, S1, and D0 states of indan: An experimental and theoretical investigation. J. Photochem. Photobiol. A Chem..

[15] Qin Z., Ma N., Ren Y., Zheng X., Yao G., Zhang X., Cui Z. (2019). Analysis of the S1 ← S0 and D0 ← S1 spectra in m-bromofluorobenzene via resonance-enhanced multiphoton ionization and slow electron velocity-map imaging spectroscopy. J. Quant. Spectrosc. Radiat. Transf..

[16] Harthcock C., Zhang J., Kong W., Mitsui M., Ohshima Y. (2017). Electronic spectra and excited-state dynamics of acridine and its hydrated clusters. J. Chem. Phys..

[17] Choi C.M., Choi D.H., Heo J., Kim N.J., Kim S.K. (2012). Ultraviolet-ultraviolet hole burning spectroscopy in a quadrupole ion trap: Dibenzo18crown-6 complexes with alkali metal cations. Angew. Chem. Int. Ed..

[18] Kang H., Féraud G., Dedonder-Lardeux C., Jouvet C. (2014). New Method for Double-Resonance Spectroscopy in a Cold Quadrupole Ion Trap and Its Application to UV-UV Hole-Burning Spectroscopy of Protonated Adenine Dimer. J. Phys. Chem. Lett..

[19] Yoo I.T., Jeong J., Eun H.J., Yun J., Heo J., Kim N.J. (2024). Conformation-Selective Ultraviolet-Ultraviolet Hole Burning Spectra of Ubiquitin Ions in a Cryogenic Ion Trap. J. Phys. Chem. Lett..

[20] Kalal B., Maity S. (2026). Non-Traditional Excited-State Deactivation in N-Containing Chromophores: A Combined Spectroscopic and Computational Study. J. Phys. Chem. Lett..

[21] Singh S.K., Mishra K.K., Sharma N., Das A. (2016). Direct Spectroscopic Evidence for an n→π* Interaction. Angew. Chem. Int. Ed. Engl..

[22] Wang S., Shi Y., Jakubek Z.J., Barnett M., Simard B., Müller-Dethlefs K., Liu C.-P., Lee Y.-P. (2002). Nonresonant two-photon mass analyzed threshold ionization and zero kinetic energy photoelectron investigation of the $\tilde{X}$^2^B_1_ ground state of CH_2_CO^+^ and CD_2_CO^+^. J. Chem. Phys..

[23] Ketkov S.Y., Tzeng S.-Y., Rychagova E.A., Lukoyanov A.N., Markin G.V., Tzeng W.-B. (2025). Intriguing methylation effects in cobaltocene revealed by high-resolution MATI spectroscopy and ab initio calculations of (η^5^-C_5_H_4_Me)_2_Co. J. Chem. Phys..

[24] Ketkov S.Y., Tzeng S.-Y., Rychagova E.A., Lukoyanov A.N., Tzeng W.-B. (2024). Effect of a single methyl substituent on the electronic structure of cobaltocene studied by computationally assisted MATI spectroscopy. Phys. Chem. Chem. Phys..

[25] Ketkov S.Y., Tzeng S.-Y., Rychagova E.A., Markin G.V., Makarov S.G., Tzeng W.-B. (2021). Laser spectroscopic and computational insights into unexpected structural behaviours of sandwich complexes upon ionization. Dalton Trans..

[26] Ketkov S.Y., Tzeng S.-Y., Wu P.-Y., Markin G.V., Tzeng W.-B. (2017). DFT-Supported Threshold Ionization Study of Chromium Biphenyl Complexes: Unveiling the Mechanisms of Substituent Influence on Redox Properties of Sandwich Compounds. Chemistry.

[27] Shi Y.J., Wang S., Jakubek Z.J., Simard B. (2004). A vacuum ultraviolet laser single-photon zero kinetic energy photoelectron spectroscopic study of the 2 E 3/2 ground electronic state of CH 3 Br +. Can. J. Chem..

[28] Lin J., Lin J.L., Tzeng W.B. (2003). Mass analyzed threshold ionization spectroscopy of N-deuterium substituted indoline cation: Isotope effect on the electronic transition, ionization and molecular vibration. Chem. Phys. Lett..

[29] Lin J., Lin J.L., Tzeng W.B. (2003). Mass analyzed threshold ionization spectroscopy of deuterium substituted N-methylaniline and N-ethylaniline cations: Isotope effect on transition energy and large amplitude vibrations. Chem. Phys..

[30] Wu L., Liu Y., Zhang C., Li S., Dixon D.A., Yang D.-S. (2012). Mass-analyzed threshold ionization of an excited state of lanthanum dioxide. J. Chem. Phys..

[31] Wu L., Zhang C., Krasnokutski S.A., Yang D.-S. (2012). Mass-analyzed threshold ionization and structural isomers of M_3_O_4_ (M = Sc, Y, and La). J. Chem. Phys..

[32] Wu L., Roudjane M., Yang D.-S. (2025). Structure and Bonding of Sc_3_C_2_ and La_3_C_2_ Clusters from MATI Spectroscopy and Theoretical Calculations. J. Phys. Chem. A.

[33] Nyambo S., Zhang Y., Yang D.-S. (2025). Reactions of La and Ce with Propylamine: Formation and Threshold Ionization of Lanthanide Imido Radicals. J. Phys. Chem. A.

[34] Ullrich S., Geppert W.D., Dessent C.E.H., Müller-Dethlefs K. (2000). Observation of Rotational Isomers I: A ZEKE and Hole-Burning Spectroscopy Study of 3-Methoxyphenol. J. Phys. Chem. A.

[35] Geppert W.D., Ullrich S., Dessent C.E.H., Müller-Dethlefs K. (2000). Observation of Rotational Isomers II: A ZEKE and Hole-Burning Spectroscopy Study of Hydrogen-Bonded 3-Methoxyphenol·Water Clusters. J. Phys. Chem. A.

[36] Wilke M., Schneider M., Wilke J., Ruiz-Santoyo J.A., Campos-Amador J.J., González-Medina M.E., Álvarez-Valtierra L., Schmitt M. (2017). Rotationally resolved electronic spectroscopy study of the conformational space of 3-methoxyphenol. J. Mol. Struct..

[37] Zheng Q., Fang T.I., Zhang B., Bih Tzeng W. (2009). Mass-analyzed Threshold Ionization Spectroscopy of Rotamers of p -ethoxyphenol Cations and Configuration Effect. Chin. J. Chem. Phys..

[38] Li C., Su H., Tzeng W.B. (2005). Rotamers of p-methoxyphenol cation studied by mass analyzed threshold ionization spectroscopy. Chem. Phys. Lett..

[39] Xu Y., Tzeng S.Y., Shivatare V., Takahashi K., Zhang B., Tzeng W.B. (2015). Identification of four rotamers of m-methoxystyrene by resonant two-photon ionization and mass analyzed threshold ionization spectroscopy. J. Chem. Phys..

[40] Qin X., Duan C., Zhao Y., Li C., Jia S. (2025). Study of Vibronic and Cationic Features of m-Diethoxybenzene via REMPI, Hole-Burning, and MATI Spectroscopy. Int. J. Mol. Sci..

[41] Li S., Zhao Y., Hu F., Jiao Y., Zhao J., Li C. (2023). The stable conformations and vibronic and cation spectroscopy of 2-ethoxybenzonitrile. J. Mol. Struct..

[42] Geppert W.D., Dessent C.E.H., Müller-Dethlefs K. (1999). ZEKE and Hole-Burning Spectroscopy of the Rotational Isomers of Resorcinol·CO. J. Phys. Chem. A.

[43] Geppert W.D., Dessent C.E.H., Ullrich S., Müller-Dethlefs K. (1999). Observation of Hydrogen-Bonded Rotational Isomers of the Resorcinol·Water Complex. J. Phys. Chem. A.

[44] Varsanyi G. (1974). Assignments of Vibrational Spectra of Seven Hundred Benzene Derivatives.

[45] Varsanyi G., Szoke S. (1969). Vibrational Spectra of Benzene Derivatives.

[46] Gerhards M., Unterberg C., Schumm S. (1999). Structure and vibrations of dihydroxybenzene cations and ionization potentials of dihydroxybenzenes studied by mass analyzed threshold ionization and infrared photoinduced Rydberg ionization spectroscopy as well as ab initio theory. J. Chem. Phys..

[47] Schneider M., Wilke M., Hebestreit M.-L., Ruiz-Santoyo J.A., Álvarez-Valtierra L., Yi J.T., Meerts W.L., Pratt D.W., Schmitt M. (2017). Rotationally resolved electronic spectroscopy of the rotamers of 1,3-dimethoxybenzene. Phys. Chem. Chem. Phys..

[48] Zhao Y., Jin Y., Hao J., Yang Y., Wang L., Li C., Jia S. (2019). Rotamers of p-isopropylphenol studied by hole-burning resonantly enhanced multiphoton ionization and mass analyzed threshold ionization spectroscopy. Spectrochim. Acta A Mol. Biomol. Spectrosc..

[49] Li S., Zhao Y., Jiao Y., Zhao J., Li C., Jia S. (2023). Vibronic and Cationic Features of 2-Fluorobenzonitrile and 3-Fluorobenzonitrile Studied by REMPI and MATI Spectroscopy and Franck-Condon Simulations. Molecules.

[50] Li N., Li S., Wang L., Wang H., Zhao J., Li C. (2022). Vibrational spectra of 2-cyanophenol cation studied by the mass analyzed threshold ionization technique. Chem. Phys. Lett..

[51] Frisch M.J., Trucks G.W., Schlegel H.B., Scuseria G.E., Robb M.A., Cheeseman J.R., Scalmani G., Barone V., Mennucci B., Petersson G.A. (2016). Gaussian 16 Revision C.01.

[52] Perdew J.P., Burke K., Wang Y. (1996). Generalized gradient approximation for the exchange-correlation hole of a many-electron system. Phys. Rev. B.

[53] Becke A.D. (1993). Density-functional thermochemistry. III. The role of exact exchange. J. Chem. Phys..

[54] Dunning T.H. (1989). Gaussian basis sets for use in correlated molecular calculations. I. The atoms boron through neon and hydrogen. J. Chem. Phys..

[55] Bauernschmitt R., Ahlrichs R. (1996). Treatment of electronic excitations within the adiabatic approximation of time dependent density functional theory. Chem. Phys. Lett..

[56] Laury M.L., Boesch S.E., Haken I., Sinha P., Wheeler R.A., Wilson A.K. (2011). Harmonic vibrational frequencies: Scale factors for pure, hybrid, hybrid meta, and double-hybrid functionals in conjunction with correlation consistent basis sets. J. Comput. Chem..

[57] Curtiss L.A., Redfern P.C., Raghavachari K. (2007). Gaussian-4 theory. J. Chem. Phys..

[58] Montgomery J.A., Frisch M.J., Ochterski J.W., Petersson G.A. (1999). A complete basis set model chemistry. VI. Use of density functional geometries and frequencies. J. Chem. Phys..

[59] Guo M., He R., Dai Y., Shen W., Li M., Zhu C., Lin S.H. (2012). Franck-Condon simulation of vibrationally resolved optical spectra for zinc complexes of phthalocyanine and tetrabenzoporphyrin including the Duschinsky and Herzberg-Teller effects. J. Chem. Phys..

[60] Santoro F., Lami A., Improta R., Bloino J., Barone V. (2008). Effective method for the computation of optical spectra of large molecules at finite temperature including the Duschinsky and Herzberg-Teller effect: The Qx band of porphyrin as a case study. J. Chem. Phys..

