# Supplementary material for: Spectroscopic Identification and Characterization of Three Rotamers of m-Ethoxyphenol: Combined REMPI, MATI, and Quantum Chemical Study
Source: Int J Mol Sci. 2026 May 7;27(10):4166. doi: 10.3390/ijms27104166 (PMC13206773; doi:10.3390/ijms27104166)
Supplement: Supplementary file 1 [file ijms-27-04166-s001.zip › ijms-4254633-supplementary.pdf]

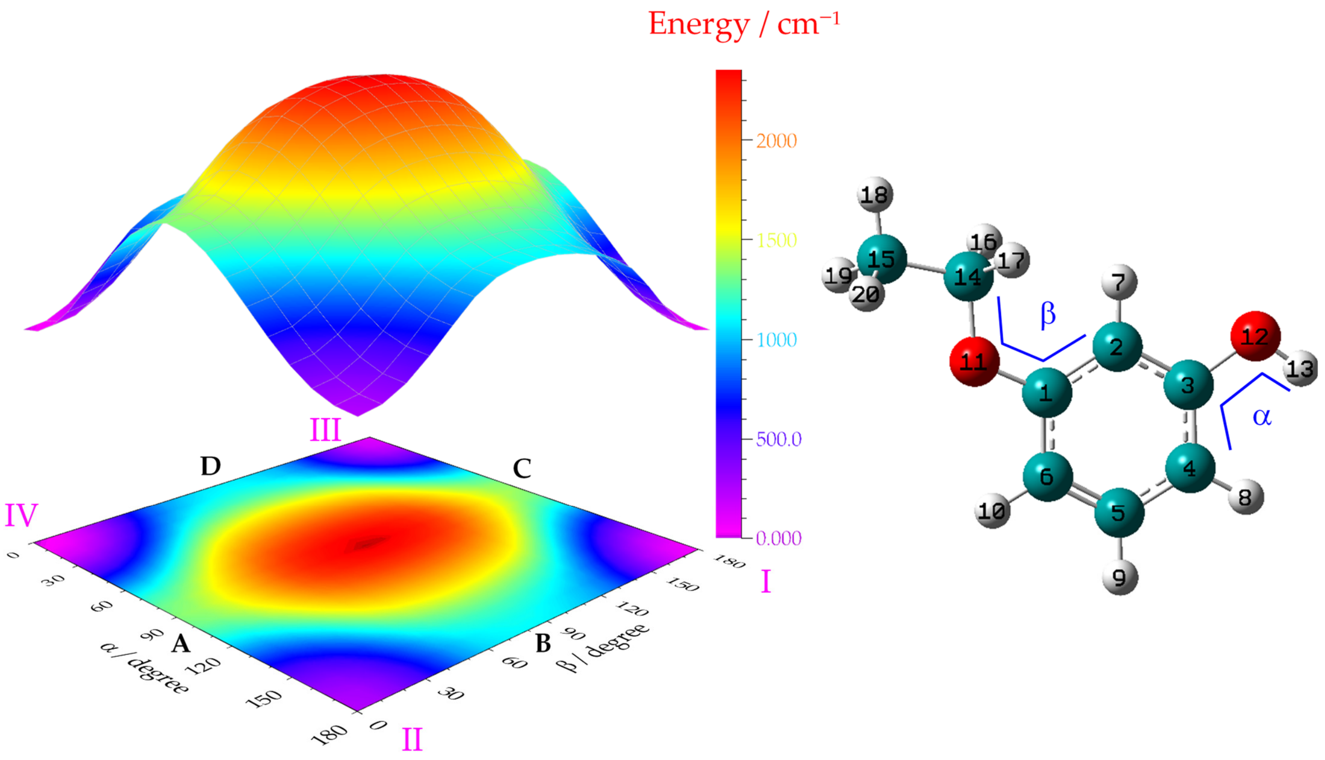

Figure S1. The potential energy surface for the electronic ground state was calculated at the B3LYP/6-311++G(d,p) level by scanning the torsional angles ∠α and ∠β. The relative energies of the four saddle points A, B, C, and D are 1337, 1104, 1365, and 1082 cm^{-1}, respectively. The energies of the four conformers I, II, III, and IV, optimized at the B3LYP/6-311++G(d,p) level (see Table 1 in the main text), are 58, 226, 90, and 0 cm^{-1}, respectively. It can be seen that the energy barriers between I and III and between II and IV exceed 1000 cm^{-1}.

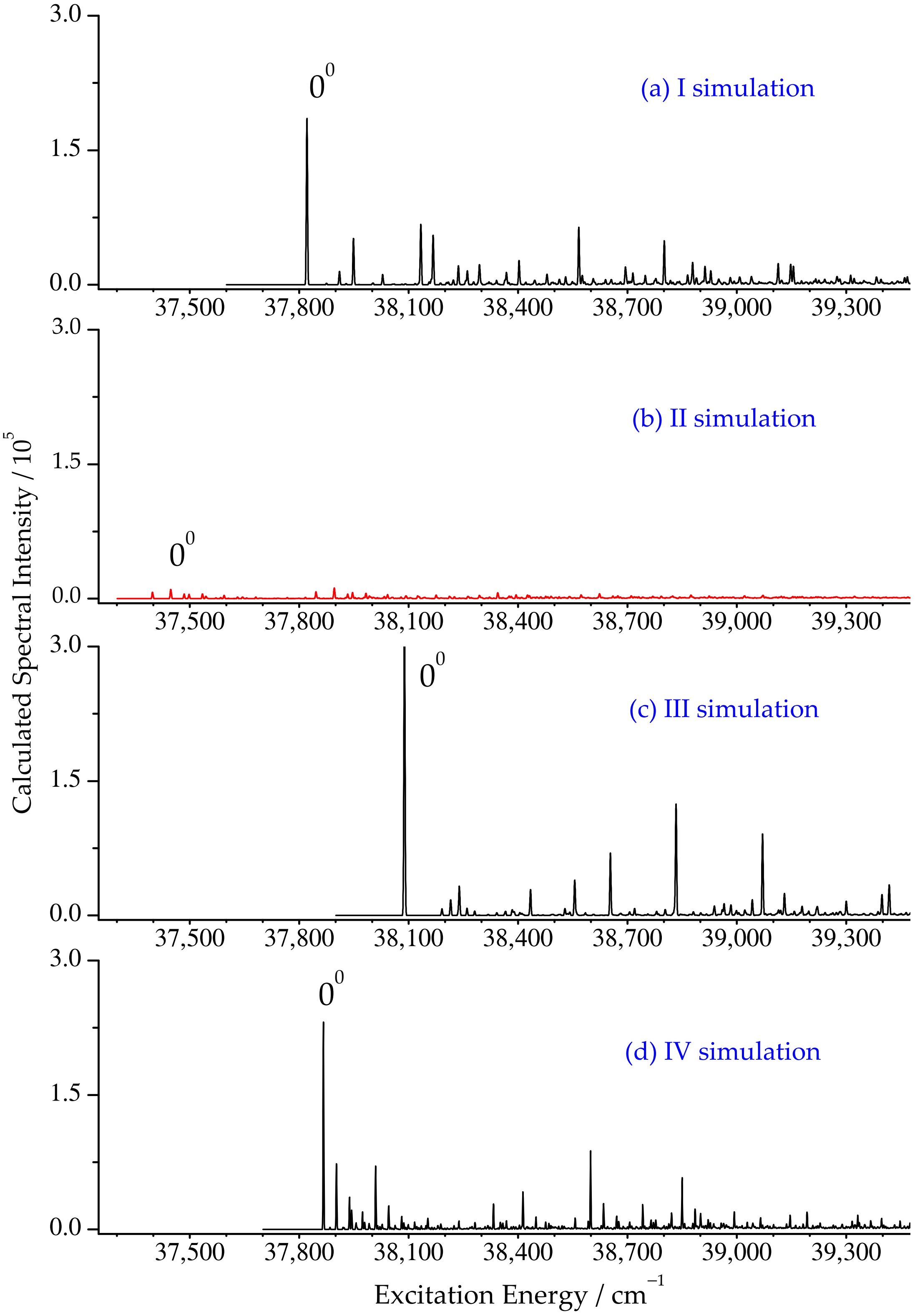

Figure S2. The calculated $S_1 \leftarrow S_0$ transition vibronic spectra for four rotamers of m-ethoxyphenol at the B3PW91/aug-cc-pVTZ level. The 0_0^0 band intensities of the four rotamers reflect the magnitude of their respective Franck–Condon factors. Horizontal and vertical axes adopt identical scales.

Table S1. Geometric parameters and the full optimized XYZ coordinates of m-ethoxyphenol rotamer IV in the S_0, S_1, and D_0 states calculated at the B3PW91/aug-cc-pVTZ, TD-B3PW91/aug-cc-pVTZ, and UB3PW91/aug-cc-pVTZ levels, respectively.

	S_0	S_1	D_0	$\Delta(S_1 - S_0)$	$\Delta(D_0 - S_1)$
Bond length (Å)					
C1-C2	1.390	1.418	1.390	0.028	–0.028
C2-C3	1.393	1.414	1.383	0.021	–0.031
C3-C4	1.387	1.410	1.438	0.023	0.028
C4-C5	1.391	1.416	1.381	0.025	–0.035
C5-C6	1.381	1.420	1.381	0.039	–0.039
C6-C1	1.398	1.411	1.444	0.013	0.033
C3-O12	1.363	1.354	1.320	–0.009	–0.034
C1-O11	1.355	1.349	1.305	–0.006	–0.044
O11-C14	1.420	1.425	1.458	0.005	0.033
C14-C15	1.509	1.508	1.503	–0.001	–0.005
Bond angle (°)					
C1C2C3	119.34	115.30	118.00	–4.04	2.70
C2C3C4	120.98	123.15	121.39	2.17	–1.76
C3C4C5	118.80	120.89	120.16	2.09	–0.73
C4C5C6	121.28	116.85	119.35	–4.43	2.50
C5C6C1	119.35	121.28	120.24	1.93	–1.04
C6C1C2	120.25	122.49	120.86	2.24	–1.63
C6C1O11	115.94	114.50	113.59	–1.44	–0.91
C4C3O12	122.49	120.76	120.80	–1.73	0.04
C3O12H13	109.11	109.31	112.81	0.18	3.52
C1O11C14	118.70	119.68	121.12	0.98	1.44
O11C14C15	107.94	107.96	107.96	0.02	0.00
Torsional angle (°)					
C4C3O12H13	0.00	–0.66	0.00	–0.66	0.66
C6C1O11C14	180.00	178.93	180	–1.03	1.07
C1O11C14C15	180.00	179.79	180	–0.17	0.19

The full optimized XYZ coordinates of S_0 state

C	-0.29520800	0.46774400	0.00003600
C	0.40648600	-0.73246100	-0.00006200
C	1.79894300	-0.70882200	-0.00009700
C	2.49287700	0.49261500	-0.00003600
C	1.77249700	1.68267300	0.00006200
C	0.39181000	1.68489700	0.00009800
H	-0.08974500	-1.69151900	-0.00011300
H	3.57702200	0.50157100	-0.00006400
H	2.30598600	2.62534000	0.00011000
H	-0.17361500	2.60674500	0.00017400
O	-1.64756500	0.55045600	0.00007600
O	2.42841600	-1.91789400	-0.00019400
H	3.37892700	-1.77764900	-0.00021000
C	-2.40427600	-0.65137200	0.00002700
C	-3.86716100	-0.27961400	0.00009600
H	-2.15280600	-1.24521500	0.88611800
H	-2.15286300	-1.24510800	-0.88615200

H	-4.48002100	-1.18235100	0.00005900
H	-4.11772800	0.30680500	0.88465400
H	-4.11778300	0.30691500	-0.88437300

The full optimized XYZ coordinates of S_1 state

C	0.29589100	0.43417100	-0.00565400
C	-0.38999800	-0.80722500	-0.02404800
C	-1.80056100	-0.71844800	0.00011300
C	-2.49607900	0.50847200	0.00846200
C	-1.79779500	1.73997900	-0.01015200
C	-0.37950500	1.67326200	0.00580500
H	0.09264600	-1.76931200	-0.03105900
H	-3.58118400	0.49144500	0.02532700
H	-2.31849200	2.68406300	-0.00772500
H	0.22442300	2.57072800	0.02578100
O	1.64117300	0.53034300	-0.00363800
O	-2.47114700	-1.89449800	0.00499100
H	-3.41801700	-1.72100200	0.02485100
C	2.43330100	-0.65449400	0.00864800
C	3.88467500	-0.24380500	0.00291300
H	2.18939200	-1.25866500	-0.87030800
H	2.18975400	-1.23852100	0.90156600
H	4.51814900	-1.13205700	0.01224900
H	4.12091800	0.33760300	-0.88886700
H	4.12263800	0.35748300	0.88083600

The full optimized XYZ coordinates of D_0 state

C	0.29877600	0.39840800	-0.00001900
C	-0.41251600	-0.79617100	0.00007000
C	-1.79401000	-0.72926900	0.00008500
C	-2.48288100	0.53330800	0.00001900
C	-1.76713400	1.71424900	-0.00006600
C	-0.38733100	1.66911200	-0.00008900
H	0.06908900	-1.76209700	0.00012800
H	-3.56714400	0.54232100	0.00003700
H	-2.28294000	2.66470200	-0.00011600
H	0.21807500	2.56610400	-0.00015500
O	1.59915800	0.50708900	-0.00004800
O	-2.46556400	-1.86567700	0.00017800
H	-3.42257800	-1.73510200	0.00019000
C	2.45372200	-0.67362600	-0.00001200
C	3.88346300	-0.21093500	-0.00011900
H	2.20760800	-1.25603100	-0.89087100
H	2.20770000	-1.25590000	0.89096000
H	4.53516100	-1.08532000	-0.00008300
H	4.10683100	0.37971700	-0.88780900
H	4.10691900	0.37985500	0.88745800

Table S2. Geometric parameters and the full optimized XYZ coordinates of m-ethoxyphenol rotamer I in the S_0, S_1, and D_0 states calculated at the B3PW91/aug-cc-pVTZ, TD-B3PW91/aug-cc-pVTZ, and UB3PW91/aug-cc-pVTZ levels, respectively.

	S_0	S_1	D_0	$\Delta(S_1 - S_0)$	$\Delta(D_0 - S_1)$
Bond length (Å)					
C1-C2	1.395	1.419	1.400	0.024	–0.019
C2-C3	1.386	1.412	1.374	0.026	–0.038
C3-C4	1.394	1.409	1.439	0.015	0.030
C4-C5	1.382	1.414	1.387	0.032	–0.027
C5-C6	1.392	1.415	1.376	0.023	–0.039
C6-C1	1.392	1.416	1.444	0.024	0.028
C1-O11	1.356	1.345	1.303	–0.011	–0.042
C3-O12	1.361	1.352	1.322	–0.009	–0.030
O11-C14	1.420	1.426	1.462	0.006	0.036
C14-C15	1.510	1.509	1.503	–0.001	–0.006
Bond angle (°)					
C1C2C3	120.03	116.00	118.74	–4.03	2.74
C2C3C4	120.56	122.83	120.77	2.27	–2.06
C3C4C5	118.69	120.61	120.2	1.92	–0.41
C4C5C6	121.88	117.66	119.91	–4.22	2.25
C5C6C1	118.78	120.79	119.71	2.01	–1.08
C6C1C2	120.06	122.11	120.66	2.05	–1.45
C6C1O11	124.54	123	122.91	–1.54	–0.09
C4C3O12	117.44	116.42	114.29	–1.02	–2.13
C3O12H13	109.38	108.99	112.5	–0.39	3.51
C1O11C14	118.5	120.48	123.06	1.98	2.58
O11C14C15	107.9	107.92	107.5	0.02	–0.42
Torsional angle (°)					
C4C3O12H13	–180	–179.98	–180	0.02	–0.02
C6C1O11C14	0	0	0	0	0
C1O11C14C15	–180	–180	–180	0	0

The full optimized XYZ coordinates of S_0 state

C	-0.29001900	-0.02389500	0.00000800
C	0.76342900	-0.93869500	0.00001200
C	2.07352800	-0.48767200	0.00000700
C	2.35282600	0.87767400	0.00000800
C	1.29816200	1.77050400	-0.00000800
C	-0.02654200	1.34338700	-0.00000100
H	0.53369100	-1.99816000	0.00000100
H	3.38116300	1.21208500	0.00000500
H	1.50598500	2.83380300	-0.00000200
H	-0.82537600	2.06973000	-0.00000400
O	-1.53234700	-0.56754500	0.00000100
O	3.13154500	-1.34438500	-0.00001300
H	2.80895700	-2.24944700	-0.00001300
C	-2.65328900	0.30405700	-0.00000500
C	-3.90133600	-0.54512500	-0.00000200
H	-2.61969300	0.94776000	-0.88623800
H	-2.61969300	0.94776800	0.88622200

H	-4.78559100	0.09410500	0.00002100
H	-3.93680800	-1.18179600	-0.88472300
H	-3.93678100	-1.18182200	0.88470200

The full optimized XYZ coordinates of S_1 state

C	-0.28400300	-0.03915400	-0.00235800
C	0.74823300	-1.01210100	0.03588200
C	2.06599100	-0.50394400	0.02212300
C	2.35623700	0.87508100	0.00359900
C	1.31568900	1.83173100	0.03120000
C	-0.01398500	1.35074700	-0.01694100
H	0.52128300	-2.03830200	0.29384800
H	3.39398800	1.17322900	-0.07432700
H	1.52439500	2.88953700	0.06540500
H	-0.83172000	2.05365600	-0.09128900
O	-1.52929800	-0.54646100	-0.02608300
O	3.12537500	-1.34359300	-0.02181900
H	2.80483800	-2.23388000	-0.21717000
C	-2.66414100	0.31645900	-0.01320600
C	-3.90198400	-0.54567300	0.00706100
H	-2.64165300	0.95247700	-0.90404600
H	-2.61570800	0.96086200	0.87001600
H	-4.79058200	0.08733600	0.01336200
H	-3.94317700	-1.18750100	-0.87326600
H	-3.92250100	-1.17585800	0.89652000

The full optimized XYZ coordinates of D_0 state

C	-0.28764300	-0.04609000	0.00004100
C	0.74936100	-0.98721300	0.00000800
C	2.04825600	-0.53914600	0.00001400
C	2.34104700	0.86985300	-0.00001300
C	1.30948400	1.79679300	-0.00000600
C	0.00188700	1.36825400	0.00002900
H	0.50105600	-2.04072700	0.00001800
H	3.38082400	1.17132800	-0.00002300
H	1.53501400	2.85456300	-0.00003200
H	-0.80897900	2.08211100	0.00002300
O	-1.50095100	-0.52019100	0.00002700
O	3.11760100	-1.31687000	-0.00002100
H	2.89147600	-2.25646000	-0.00004800
C	-2.69008300	0.33097500	-0.00004300
C	-3.89195800	-0.57147600	-0.00000600
H	-2.65057700	0.95744100	-0.89282900
H	-2.65059200	0.95753900	0.89267600
H	-4.79221200	0.04421400	-0.00004700
H	-3.91064900	-1.20265800	-0.88769600
H	-3.91066600	-1.20256100	0.88775300

Table S3. Geometric parameters and the full optimized XYZ coordinates of m-ethoxyphenol rotamer III in the S_0, S_1, and D_0 states calculated at the B3PW91/aug-cc-pVTZ, TD-B3PW91/aug-cc-pVTZ, and UB3PW91/aug-cc-pVTZ levels, respectively.

	**S_0**	**S_1**	**D_0**	**$\Delta(S_1 - S_0)$**	**$\Delta(D_0 - S_1)$**
Bond length (Å)					
C1-C2	1.393	1.418	1.398	0.025	–0.020
C2-C3	1.384	1.409	1.374	0.025	–0.035
C3-C4	1.395	1.412	1.439	0.017	0.027
C4-C5	1.384	1.417	1.388	0.033	–0.029
C5-C6	1.389	1.419	1.376	0.030	–0.043
C6-C1	1.395	1.416	1.446	0.021	0.030
C1-O11	1.356	1.346	1.303	–0.010	–0.043
C3-O12	1.362	1.354	1.323	–0.008	–0.031
O11-C14	1.420	1.424	1.462	0.004	0.038
C14-C15	1.509	1.509	1.503	0	–0.006
Bond angle (°)					
C1C2C3	119.86	116.24	118.92	–3.62	2.68
C2C3C4	120.57	122.62	120.52	2.05	–2.10
C3C4C5	118.89	120.69	120.41	1.80	–0.28
C4C5C6	121.59	117.62	119.85	–3.97	2.23
C5C6C1	118.81	120.65	119.58	1.84	–1.07
C6C1C2	120.29	122.18	120.72	1.89	–1.46
C6C1O11	124.29	123.23	122.74	–1.06	–0.49
C4C3O12	122.16	120.72	120.93	–1.44	0.21
C3O12H13	109.16	109.28	112.83	0.12	3.55
C1O11C14	118.61	120.45	123.22	1.84	2.77
O11C14C15	107.9	107.92	107.47	0.02	–0.45
Torsional angle (°)					
C4C3O12H13	–0.02	–0.06	0	–0.04	0.06
C6C1O11C14	0	0	0	0	0
C1O11C14C15	–180	–179.99	–180	0.01	–0.01

The full optimized XYZ coordinates of S_0 state

C	0.29438600	-0.03341400	0.00001200
C	-0.75273100	-0.95210200	-0.00012700
C	-2.06232600	-0.50417400	-0.00016500
C	-2.34485000	0.86158700	-0.00006300
C	-1.29382300	1.76168500	0.00007900
C	0.02877400	1.33608300	0.00011800
H	-0.53905000	-2.01223100	-0.00021100
H	-3.37188200	1.20928500	-0.00009600
H	-1.50538500	2.82410200	0.00016000
H	0.82653900	2.06355100	0.00022700
O	1.53931800	-0.56995700	0.00004000
O	-3.04468200	-1.44782900	-0.00031400
H	-3.90079200	-1.01184300	-0.00026800
C	2.65713700	0.30571600	0.00018700
C	3.90819300	-0.53891900	0.00018200
H	2.62174100	0.94933500	0.88649200
H	2.62184500	0.94950000	-0.88600200

H	4.79043600	0.10315000	0.00033800
H	3.94537200	-1.17577400	0.88456200
H	3.94552200	-1.17555100	-0.88435100

The full optimized XYZ coordinates of S_1 state

C	-0.29424000	-0.04346100	0.00016500
C	0.73717400	-1.01639300	0.00074000
C	2.05716800	-0.52464300	-0.00004700
C	2.35522700	0.85561100	-0.00011000
C	1.31698800	1.81971700	0.00053200
C	-0.02039400	1.34603800	-0.00020200
H	0.51937800	-2.07200100	-0.00000200
H	3.39423300	1.16852800	-0.00033500
H	1.53274400	2.87640200	0.00041000
H	-0.83466000	2.05586100	-0.00122300
O	-1.54194700	-0.54957400	0.00010300
O	3.04890600	-1.44639100	-0.00024100
H	3.90041000	-0.99722000	-0.00140400
C	-2.67212600	0.31664900	-0.00033200
C	-3.91377700	-0.54042600	0.00001900
H	-2.63496900	0.95701300	-0.88768400
H	-2.63505200	0.95781300	0.88643600
H	-4.80009500	0.09585700	-0.00049700
H	-3.94677000	-1.17709600	-0.88445100
H	-3.94701100	-1.17599400	0.88527300

The full optimized XYZ coordinates of D_0 state

C	0.29028200	-0.04957600	0.00001300
C	-0.74278700	-0.99086200	-0.00013200
C	-2.04385600	-0.54933300	-0.00016900
C	-2.33754900	0.85944800	-0.00006000
C	-1.30935300	1.79128900	0.00008400
C	-0.00081500	1.36699400	0.00012300
H	-0.51316700	-2.04710100	-0.00021300
H	-3.37210400	1.18464600	-0.00009200
H	-1.53933400	2.84805200	0.00016500
H	0.80760300	2.08357500	0.00023400
O	1.50530400	-0.51922900	0.00004300
O	-3.01629400	-1.44697500	-0.00030700
H	-3.89476600	-1.04726900	-0.00032300
C	2.69343000	0.33260500	0.00018900
C	3.89548300	-0.56967200	0.00017500
H	2.65349200	0.95885200	0.89314800
H	2.65359400	0.95901500	-0.89266100
H	4.79558000	0.04617900	0.00028600
H	3.91395700	-1.20092200	0.88773000
H	3.91406200	-1.20075700	-0.88749500
